# Acupuncture Regulates Serum Differentially Expressed Proteins in Patients with Chronic Atrophic Gastritis: A Quantitative iTRAQ Proteomics Study

**DOI:** 10.1155/2021/9962224

**Published:** 2021-06-14

**Authors:** Feng Li, Bai Yang, Yanan Liu, Tianying Tang, Cun Wang, Mei Li, Siyi Lv, Qin Qi, Huirong Liu, Zheng Shi, Huangan Wu, Xiaomei Wang

**Affiliations:** ^1^Yueyang Clinical Medical College, Shanghai University of Traditional Chinese Medicine, Shanghai, China; ^2^Yueyang Hospital of Integrated Traditional Chinese and Western Medicine, Shanghai University of Traditional Chinese Medicine, Shanghai, China; ^3^Key Laboratory of Acupuncture-Moxibustion and Immunological Effects, Shanghai University of Traditional Chinese Medicine, Shanghai, China; ^4^Shanghai Research Institute of Acupuncture and Meridian, Shanghai University of Traditional Chinese Medicine, Shanghai, China

## Abstract

**Objective:**

To identify differentially expressed proteins (DEPs) in sera of patients with chronic atrophic gastritis (CAG) using isobaric tags for relative and absolute quantitation (iTRAQ) and to explore acupuncture's mechanism in CAG.

**Methods:**

Peripheral sera from 8 healthy volunteers (HC), 8 chronic nonatrophic gastritis (NAG) patients, 8 CAG patients, and 8 CAG patients who underwent acupuncture treatment (CAG + ACU) were collected followed by labeling with iTRAQ reagent for protein identification and quantification using two-dimensional liquid chromatography-tandem mass spectrometry (2D-LC-MS/MS). Representative DEPs were selected through bioinformatics, and proteins were verified by enzyme-linked immunosorbent assay (ELISA).

**Results:**

A total of 4,448 unique peptides were identified, corresponding to 816 nonredundant proteins. A 1.4-fold difference was used as the threshold. Compared with the HC group, 75 and 106 DEPs were identified from CAG and NAG groups, respectively. Compared with the CAG group, 110 and 66 DEPs were identified from the NAG and CAG + ACU groups, respectively. The DEPs were mainly involved in protein binding and the Notch signaling pathway-related proteins, and the upregulated proteins included actin-binding proteins (thymosin beta-4, tropomyosin-4, profilin-1, transgelin-2), while the downregulated proteins included Notch2 and Notch3. After acupuncture, the expression of these proteins in CAG patients was less differentiated from that in healthy people. The level of the above 6 proteins were verified by ELISA, and the results were similar to the results of iTRAQ analysis.

**Conclusions:**

Actin-binding proteins and Notch signaling pathway-related proteins were correlated with the development and progression of CAG and thus are potential diagnostic markers for CAG. Acupuncture may play a role in regulating actin-binding proteins and Notch signaling pathway-related proteins to play a therapeutic role in CAG.

## 1. Introduction

Chronic gastritis is characterized as either chronic atrophic gastritis (CAG) or nonatrophic gastritis (NAG), of which CAG includes gland atrophy and intestinal metaplasia and is recognized as a precancerous lesion [1, 2]. Correa's cascade summarizes the evolution of chronic gastritis as NAG ⟶ CAG ⟶ gastric intestinal metaplasia of the gastric mucosa ⟶ gastric mucosal dysplasia ⟶ gastric cancer [3]. Global Cancer Statistics in 2018 showed that gastric cancer was the fifth most common malignant tumor and the third leading cause of cancer death globally [4]. Hence, as CAG is a precancerous lesion, its early diagnosis and treatment are particularly important to prevent the occurrence of gastric cancer.

In clinical practice, most CAG patients have no obvious symptoms. When symptoms are present, they manifest primarily as dyspepsia, epigastric pain, abdominal distension, and other nonspecific symptoms. The diagnosis of CAG mainly relies on gastroscopy and gastric mucosal biopsy [1]. However, for various reasons, gastroscopy is not a popular procedure for CAG diagnosis in many countries. In recent years, some researchers have proposed a combined analysis of serum gastrin-1, pepsinogen I, pepsinogen II, and their ratios for CAG diagnosis [5], but studies have shown that serum gastrin-17 levels are not significantly correlated with the degree of antral atrophy and cannot be used as the gold standard of CAG diagnosis and treatment [6]. In addition, another therapeutic regimen for *Helicobacter pylori* (HP)-positive CAG involves using triple therapy (i.e., proton pump inhibitor or bismuth plus 2 antibiotics) to eradicate HP in order to improve the clinical symptoms of CAG; in addition, gastric mucosal-protective agents, digestive enzyme inhibitors, and gastrointestinal motility drugs can be used according to the symptoms.1 However, some studies have shown that long-term application of proton pump inhibitors in HP-infected subjects causes an increased risk of gastric cancer and is related to drug dosage and time factors [7]. A growing number of studies have shown that acupuncture treatment holistically improves the clinical symptoms of CAG patients with low cost and no toxic side effects [8–10]. In recent years, research on the mechanism of acupuncture and moxibustion in CAG treatment has mainly focused on immune function, mucosal protection, gastrointestinal hormones, and metabolic changes [11–13]. However, the effect of acupuncture on differentially expressed proteins (DEPs) in the serum of CAG patients remains unknown.

Isobaric tags for relative and absolute quantitation (iTRAQ) is a high-throughput screening technology that has been extensively used in quantitative proteomics in recent years. Its characteristics include high sensitivity, strong separation ability, and high throughput. Furthermore, iTRAQ has often been used to screen for and identify biomarkers [14]. Recent studies have shown that iTRAQ can identify DEPs in gastric cancer, such as new molecular biomarkers (e.g., glutaminase 1, *γ*-glutamyl cyclotransferase, calcium-binding proteins, and granulin) [15–17]. Nevertheless, there are few studies on the pathogenesis of CAG that examine DEPs. Thus, screening for serum markers of CAG may provide vital knowledge about CAG pathogenesis and the prevention of gastric cancer.

Our previous clinical studies have shown that acupuncture, one of the therapies in traditional Chinese medicine, significantly improves clinical symptoms in CAG patients, such as stomachache, abdominal distension, anxiety, and depression, with a total effective rate of 84.38%, improving their quality of life [18, 19]. On this basis, we chose 8 effective patients in acupuncture treatments using iTRAQ and liquid chromatography coupled with tandem mass spectrometry (LC-MS/MS) to conduct relative quantitative analysis of proteins to determine the targets of acupuncture treatment in CAG. This study also used functional annotation, pathway enrichment, and other bioinformatic analysis methods to explore the pathogenesis of CAG and the related mechanism of acupuncture treatment.

## 2. Methods

### 2.1. Research Subjects

This study included 24 research subjects, consisting of healthy volunteers (*n* = 8, A group, labeled 114, 18–70 years old), NAG patients (*n* = 8, B group, labeled 115, 18–70 years old), CAG patients (*n* = 8, C group, labeled 116, 18–70 years old), and CAG patients who underwent acupuncture treatment (*n* = 8, D group, labeled 117, 18–70 years old). Healthy volunteers were those with normal gastric mucosa on gastroscopy, while the other patients had mild and moderate CAG or NAG and were treated at the medical outpatient clinic of the Shanghai Research Institute of Acupuncture and Meridian, Shanghai, China, from May 2016 to June 2019. The diagnostic criteria were in accordance with the Consensus on Chronic Gastritis in China issued by the Chinese Society of Gastroenterology [20]. The mucosa specimens of gastric were taken under gastroscopy, and the degree of atrophy/intestinal metaplasia were evaluated by the OLGA (Operative Link for Gastritis Assessment) staging system [21].

Inclusion criteria were (1) age between 18 and 70 years, no gender limitation; (2) the pathological diagnosis was consistent with chronic gastritis on gastroscopy; and (3) understand and agree to participate in this study and sign the informed consent. Exclusion criteria were (1) individuals that fell outside the age range; (2) patients diagnosed with schizophrenia, major depression, and other psychiatric disorders; (3) patients previous diagnosis of gastric cancer, massive gastrointestinal bleeding and gastric perforation; (4) patients with hypertension, diabetes, heart disease, renal dysfunction, liver disease, or malignant diseases; (5) pregnant women, breastfeeding women, and women planning to be pregnant; (6) patients who had participated in other clinical trials within 1 month; and (7) the selected acupoint area has skin diseases, limb deformity, and other conditions that cannot be treated by acupuncture.

This study was approved by the Ethics Committees of the Shanghai Yueyang Integrated Traditional Chinese Medicine and Medicine Hospital affiliated to the Shanghai University of Traditional Chinese Medicine (Approval number: 2016-028) and has been registered in the Chinese Clinical Trial Registration Center (http://www.chictr.org.cn) (registration number: ChiCTR1900026044). All participants signed the informed consent and were diagnosed via gastroscopy and histopathological examinations of gastric mucosa biopsy specimens. Basic clinical parameters of healthy volunteers, NAG patients, and CAG patients before treatment are displayed in Table 1.

8 CAG patients were treated with acupuncture at the selected acupoints: Zhongwan (RN12), Neiguan (PC6, bilateral), and Zusanli (ST36, bilateral), according to the standards for acupoint selection established by the World Health Organization, the *Global Standard Acupuncture Point Locations* [22]. The needles used in this study were 0.3 × 40 mm disposable sterile acupuncture needles (Hwato; Suzhou Medical Appliance Factory, Jiangsu Province, China). The acupuncture depth was 15–20 mm, and the acupuncture technique was straight, flattening, and replenishing. The needles remained inserted in the patients for 20 min after deqi (feeling the sensation of soreness, numbness, distension, and heaviness). Acupuncture was performed 3 times per week, 10 times per treatment course, for a total of 6 courses by the same acupuncturist. Clinical features of CAG patients before and after acupuncture treatment along with clinical outcomes are displayed in Table 2.

### 2.2. Specimen Collection

Morning fast peripheral blood (10 mL) from each group was collected after enrolling, and 10 mL blood from the D group was collected after 6 courses' acupuncture treatment. The samples were kept at room temperature for 30 min before centrifugation at 3,000 rpm for 15 min in a 4°C centrifuge (Fresco17; Thermo Fisher Scientific, Waltham, MA, USA) to collect supernatants that were aliquoted in cryopreservation tubes, labeled, and stored in a −80°C freezer for later experiments.

### 2.3. Removing High-Abundance Proteins

After thawing the frozen serum samples on ice, 50 *μ*L of each serum sample was removed, and samples from the same group were pooled before centrifugation at 13,200 rpm for 10 min to collect and transfer the middle layer of serum into a new tube. The samples were sorted and labeled independently as A (HC), B (NAG), C (CAG), or D (CAG + ACU) group according to the sample grouping information. First, the samples were subjected to the Agilent-Human 14 multiple affinity removal system (MARS human-14; Agilent technologies, Santa Clara, CA, USA) to remove 14 high-abundance serum proteins, including albumin, immunoglobulin (Ig)G, antitrypsin, IgA, transferrin, haptoglobin, fibrinogen, *α*2-macroglobulin, *α*1-acid glycoprotein, IgM, apolipoprotein AI, apolipoprotein AII, complement protein C3, and trans-thyroglobulin, followed by quantifying the protein concentrations in each group using a bicinchoninic acid (BCA) assay kit (Beyotime, Shanghai, China) and subsequently adjusting the concentration to 2 mg/mL in each group.

### 2.4. Filter-Aided Sample Preparation (FASP) for Whole Protein Cleavage

A protein sample (100 *µ*L) from each group was added with dithiothreitol and incubated in a 56°C water bath for 1 h followed by adding 100 *µ*L iodoacetamide for further incubation at room temperature in the dark for 40 min. After reductive alkylation, the samples were transferred to 10 K ultrafiltration tubes and centrifuged at 12,000 rpm for 10 min to discard the bottom solution of the collection tube. Each supernatant sample was added to 100 *µ*L lysis buffer (triethylammonium bicarbonate) in an ultrafiltration tube. Each sample was washed 4 times followed by adding 40 *µ*L trypsin to the ultrafiltration tube for enzymatic hydrolysis in a 37°C incubator overnight. On the next day, the collection tubes were renewed and centrifuged at 12,000 rpm for 10 min. Each sample was diluted with lysis buffer to 100 *µ*L. Enzymatic hydrolysates (5 *µ*L for each sample) were collected for LC-MS analysis to calculate the number of missed pancreatin sites.

### 2.5. iTRAQ Labeling

The iTRAQ Reagent-Multiplex Buffer Kit (ABSCIEX, Darmstadt, Germany) was equilibrated at room temperature for 20 min followed by centrifuging the reagents at 12,000 rpm for 10 min. Next, 50 *µ*L isopropanol was added and mixed well using a vortex mixer. The iTRAQ reagent was added into a 50 *µ*L sample from each group in a new centrifuge tube and labeled. The reaction was stopped after 3 h of incubation at room temperature (22 ± 2°C). The labeled samples were mixed and freeze-dried.

### 2.6. Offline High-Performance Liquid Chromatography (HPLC) Preclassification

Each freeze-dried sample was reconstituted with 30 *μ*L of 20 mM ammonium formate, vortexed, and centrifuged at 12,000 rpm for 10 min. Subsequently, 25 *μ*L of supernatant was collected and subjected to high-pH reversed-phase chromatography for preclassification in the HPLC system (EASY-nLC 1000; Thermo Fisher Scientific). A total of 10 tubes of sample solution were obtained after screening and were centrifuged and freeze-dried.

### 2.7. LC-MS/MS Analysis

The separated and lyophilized peptide samples were redissolved in Buffer A (acetonitrile:water:formic acid = 2 : 98 : 0.1), dissolved on a vortex mixer, moved to a sample bottle, and subsequently subjected to MS analysis. Online Nano-RPLC was performed via the Easy-nLC 1000 system (Thermo Fisher Scientific). The dissolved sample was loaded onto a trap column (PepMap100, C18 3 *μ*m 75 *μ*m × 20 mm NanoViper, Thermo Fisher Dionex ion chromatography; Thermo Fisher Scientific) at a flow rate of 2 µL/min followed by rinse desalination for 10 min. The analytical column was a C18 reverse-phase chromatography column (PepMap100, C18 2 *μ*m 75 *μ*m × 150 mm NanoViper, Thermo Fisher Dionex ion chromatography). The gradient used in the experiment increased the mobile phase B from 5% to 35% within 70 min.

The Q-Exactive MS system (Thermo Fisher Scientific) combined with a nanospray ion source (Thermo Fisher Scientific) was used. The spray voltage was 1.6 kV, and the temperature of the capillary tube was 250°C. The MS scanning mode was set as the data-dependent acquisition mode (Data Dependent Analysis, DDA). Twenty fragment maps were collected after each full scan. The full-scan resolution was 70,000, the MS/MS resolution was 17,500, the precursor ion scan range was 300–1,800 m/z, and the collision energy was 27% higher energy C-trap dissociation.

### 2.8. Data Processing

The original data were converted into *mgf* files using the Proteome Discoverer (version 1.3; Thermo Fisher Scientific) software, and the data retrieval and analysis were performed in the UniProt human protein reference database (148,986 sequences). Subsequently, the target-decoy database was used to evaluate the results. For protein identification, a mass tolerance of 20 ppm was permitted for intact peptide masses and 0.05 Da for fragmented ions. One missed cleavage was allowed in the trypsin digests. The variable modifications were oxidation (M) and iTRAQ 4plex (Y); the fixed modifications were carbamidomethyl (C), iTRAQ 4plex (N-term), and iTRAQ 4plex (K). The charge state of peptides was set to +2 and +4.

The protein identification was grouped according to the peptide tags in all samples. The protein ratio was calculated according to the specific peptides, and the protein identification confidence was set to 95% (i.e., Prot Score > 1.3) to search and remove the false-positive proteins in the decoy database (FDR < 1%). The threshold was protein difference fold, which reached 1.4-fold (ratio ≥ 1.4 or ≤ 0.714). In addition, Gene Ontology (GO) analysis, Kyoto Encyclopedia of Genes and Genomes (KEGG) pathway enrichment analysis, protein interaction network analysis, clustered heap map analysis, expression trend analysis, and similarity and difference analysis of DEPs between groups were conducted to discover the functional properties of these DEPs and their relevance to the research goal. This analysis was used to identify the representative proteins for subsequent ELISA detection.

### 2.9. Validation of the Candidate Proteins

Six representative serum DEPs, including thymosin beta-4, profilin-1, tropomyosin-4, transgelin-2, Notch2, and Notch3, were selected according to the results of bioinformatics analysis. The DEPs were quantified using ELISA kits purchased from Shanghai Lengton Bioscience (Shanghai, China). A total of 32 samples were applied to determine the concentration of these proteins in each serum of the validation set from 4 groups (8 HC, 8 NAG, 8 CAG, 8 CAG + ACU). Each sample was performed in duplicate according to the ELISA manufacturer's instructions. The enzymatic reaction substrate was added to produce a chromogenic reaction. The chromogenic differences between the experimental group and the control group were compared to calculate the content of proteins.

### 2.10. Statistical Analysis

SPSS software (version 19; IBM SPSS Statistics, USA) was used for statistical analysis. Measurement data with normal distribution were shown as mean ± standard deviation, and one-way ANOVA was used. The data with non-normal distributions were showed as median (P25, P75), and nonparametric tests were used. Fisher's exact test was used to classify the classification variables. The significance level adopted was 0.05.

## 3. Results

### 3.1. Protein Identification from Plasma Samples

**Figure 1:**
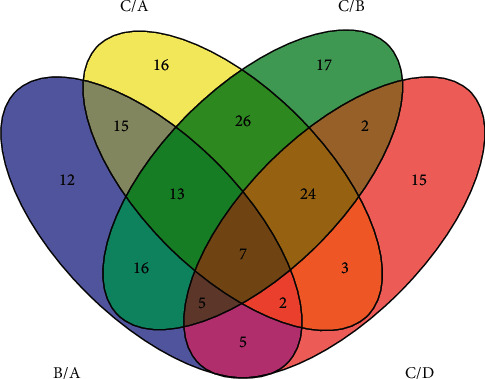
Differences and similarities of the four identified proteins were analyzed according to the protein uniprot number. A: healthy controls; B: NAG group; C: CAG group; D: CAG + ACU group.

After LC-MS/MS analysis, a total of 99,509 spectra were matched to 6,628 peptides (including 4,448 unique peptides). These identified peptides were matched to 816 proteins. Using a protein difference ratio of 1.4-fold as the threshold (ratio ≥ 1.4 or ratio ≤ 0.714) and using healthy volunteers as the HC group, a total of 178 DEPs were identified in the NAG, CAG, and CAG + ACU groups (Supplementary File 1).  Table 3 and Figure 1 summarize the DEP statistics.

### 3.2. GO Enrichment Analysis

**Figure 2:**
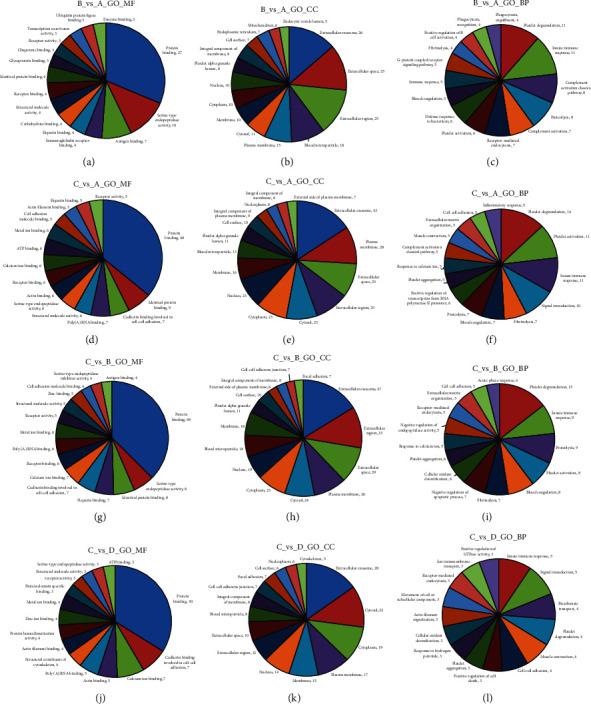
Classification of the identified proteins by GO database. A: healthy controls; B: NAG group; C: CAG group; D: CAG + ACU group. MF: molecular function; CC: cellular component; BP: biological process. (a) B vs. A in MF; (b) B vs. A in CC; (c) B vs. A in BP; (d) C vs. A in MF; (e) C vs. A in CC; (f) C vs. A in BP; (g) C vs. B in MF; (h) C vs. B in CC; (i) C vs. B in BP; (j) C vs. D in MF; (k) C vs. D in CC; (l) C vs. D in BP.

The 4 groups of DEPs were classified according to the molecular function (MF), cellular component (CC), and biological process (BP) of each protein via the GO database followed by using Fisher's exact test (*p* < 0.05) to obtain the protein function classifications that were significantly related to CAG (Figure 2). In MF, DEPs were mainly involved in protein binding in NAG, CAG, and CAG + ACU groups. In CC, DEPs were mainly involved in extracellular exosome, and in BP, DEPs were mainly involved in platelet degranulation and innate immune response in three groups. These results suggested that protein binding and immune system response were related to CAG.

### 3.3. KEGG Pathway Enrichment Analysis

**Figure 3:**
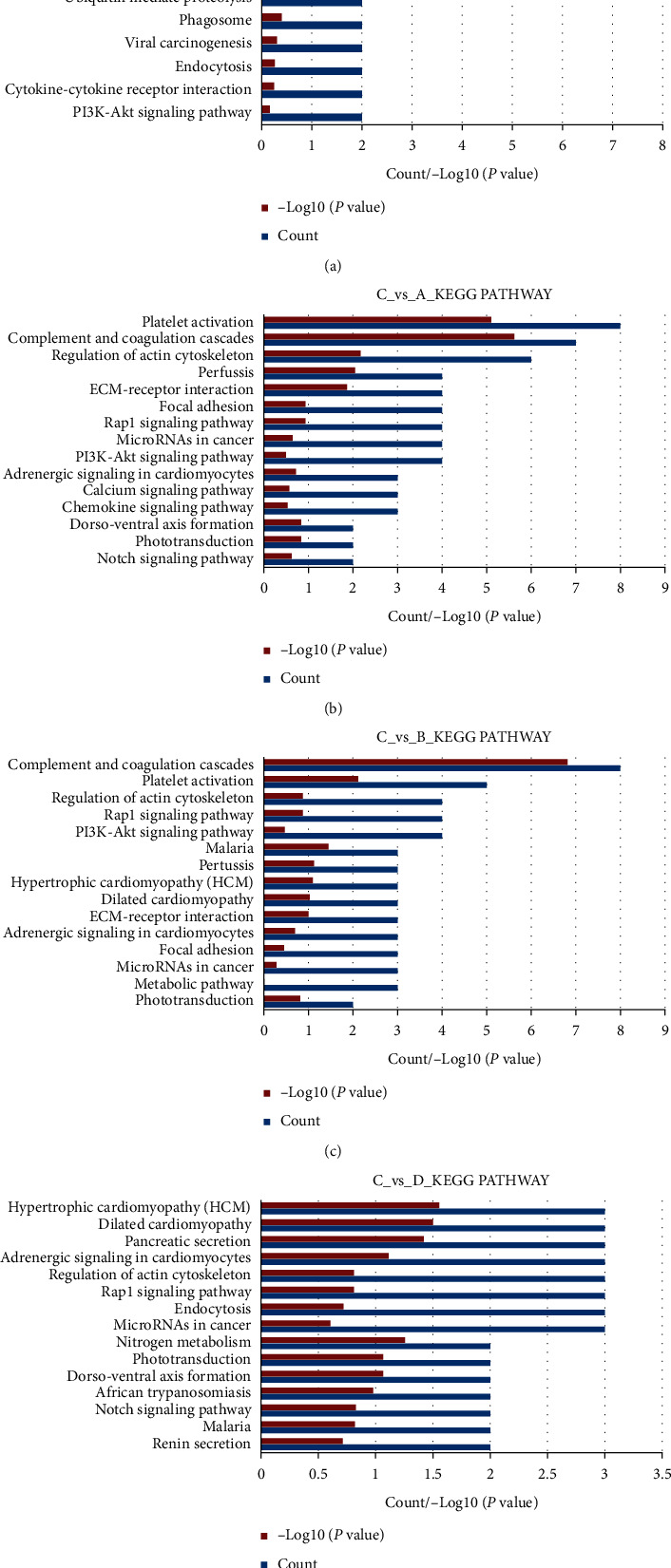
Classification of the identified proteins by KEGG database. (a) The eleven most significant KEGG pathways in NAG group vs. healthy controls. (b) The fifteen most significant KEGG pathways in CAG group vs. healthy controls. (c) The fifteen most significant KEGG pathways in CAG group vs. NAG group. (d) The fifteen most significant KEGG pathways in CAG group vs. CAG + ACU group.

Based on the KEGG database (http://www.genome.jp/kegg/), the DEPs were enriched and analyzed. The enriched pathways are shown in Figure 3; results showed that the occurrence and progression of NAG were mainly involved in the complement system, platelet activation, and chemokine signaling pathway. In addition to the above signaling pathways, CAG also participated in actin cytoskeleton regulation and Notch signaling pathways. Acupuncture may play a therapeutic role by regulating the actin cytoskeleton and Notch signaling pathway.

### 3.4. Protein-Protein Interaction (PPI) Network Analysis

**Figure 4:**
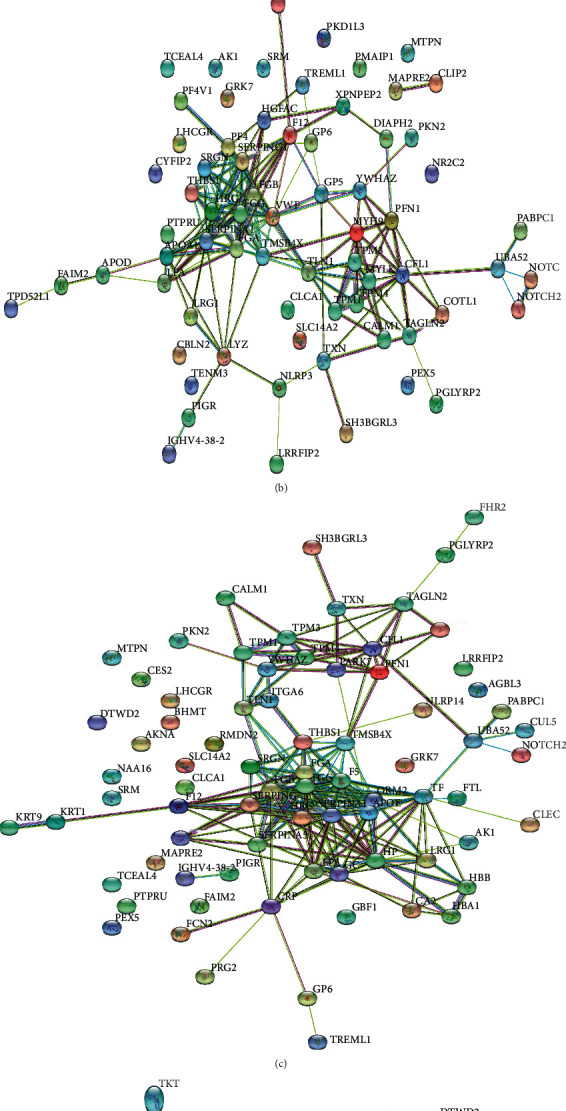
Association networks of dysregulated proteins. (a) The protein-protein interaction networks in NAG group vs. healthy controls. (b) The protein-protein interaction networks in CAG group vs. healthy controls. (c) The protein-protein interaction networks in CAG group vs. NAG group. (d) The protein-protein interaction networks in CAG group vs. CAG + ACU group.

Based on the STRING database (https://string-db.org/) and Cytoscape software (V3.6.0), the 4 groups of DEPs were analyzed by PPI, and the PPI network diagrams were prepared (Figure 4). The PPI analysis showed that the DEP interaction network of NAG mainly involved complement and coagulation cascades (APOA1, APOE, ORM2, FGA, SERPINA1, HP, etc.). The DEP interaction network of CAG also involved the regulation of the actin cytoskeleton (TPM1, TPM3, TPM4, TMSB4X, MYLK, MYH9, CFL1, PFN1, TAGLN2, COTL1, etc.). Thus, acupuncture may exert its therapeutic role on CAG by regulating these pathways.

### 3.5. Clustered Heatmap Analysis

**Figure 5:**
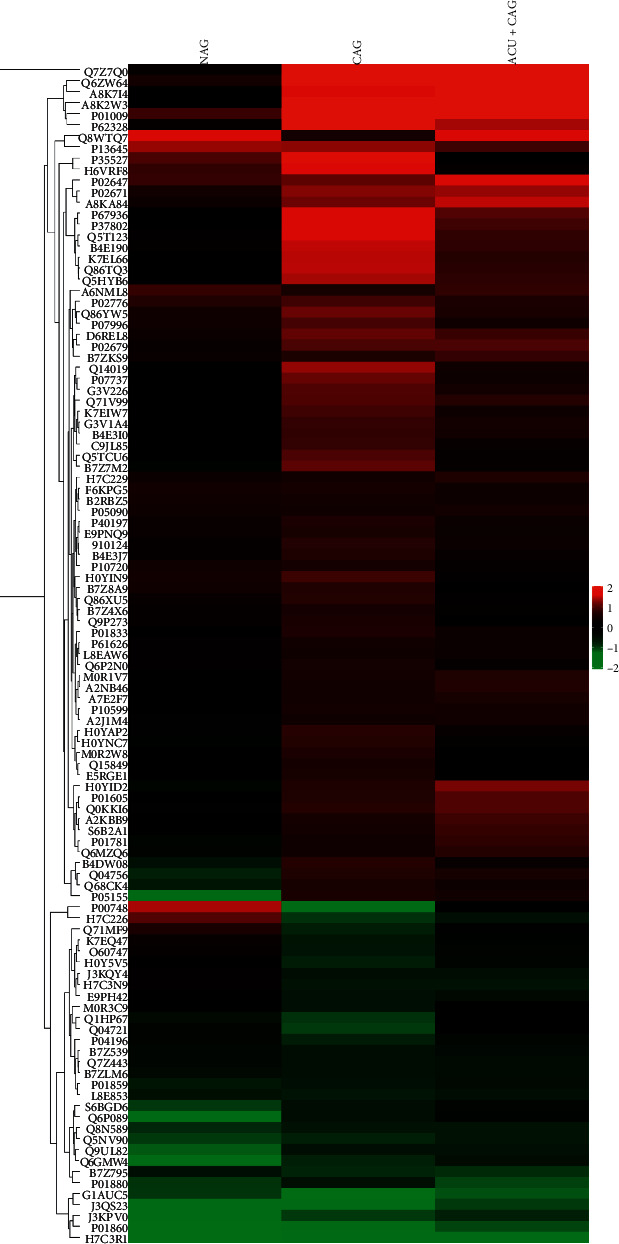
Clustered heatmap analysis of the differential proteins in NAG, CAG, and CAG + ACU groups. Each column represents the protein information of one group of samples, and each row represents the relative expression level of each protein. Red represents significantly upregulated proteins, green represents significantly downregulated proteins, and black represents no significant difference in proteins.

The expression levels of DEPs in different groups were displayed in different colors in the form of Log_2_ expression. As shown in Figure 5, most of the proteins in the 3 groups (NAG, CAG, and CAG + ACU) were different. In the clustered heatmap, protein expression in the NAG group is mostly shown in black and demonstrates no significant differences from the HC group. Most proteins showed upregulated expression in the CAG group (depicted in red), showing significant differences from the HC group. The expression levels of most proteins in the CAG + ACU group tended to be similar to those in the NAG group. Among them, thymosin beta-4, profilin-1, tropomyosin-4, transgelin-2, Notch2, and Notch3 showed a strong trend in three groups.

### 3.6. Trend Analysis

**Figure 6:**
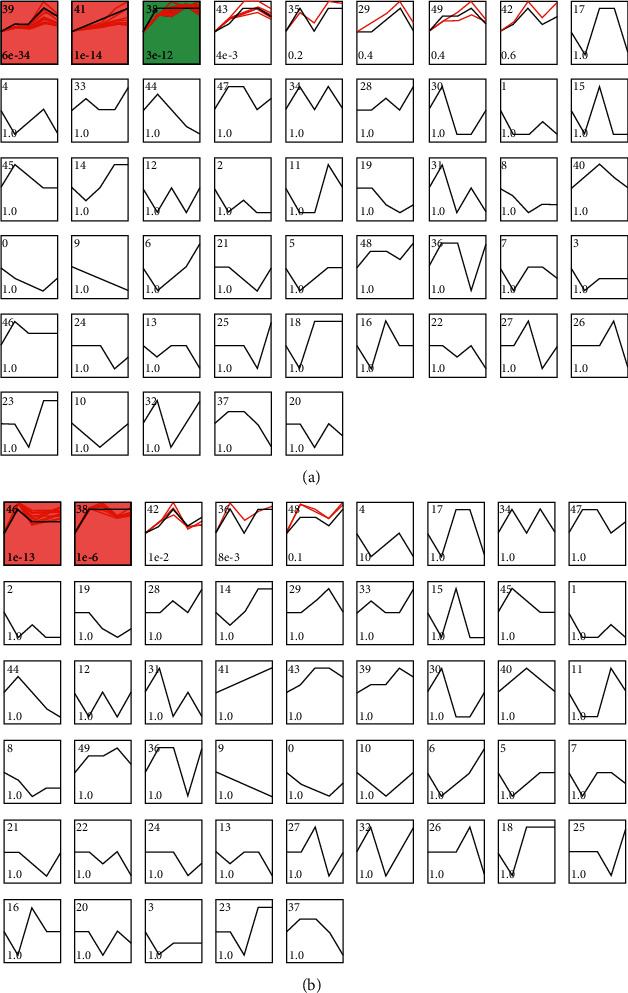
The expression patterns of 106 proteins analyzed by model profile, and fifty model profiles were used to summarize. Each box represents a model expression profile. The upper number in the profile box is the model profile number, and the lower one is the *p* value. (a) Upregulated protein tendency; (b) downregulated protein tendency.

**Figure 7:**
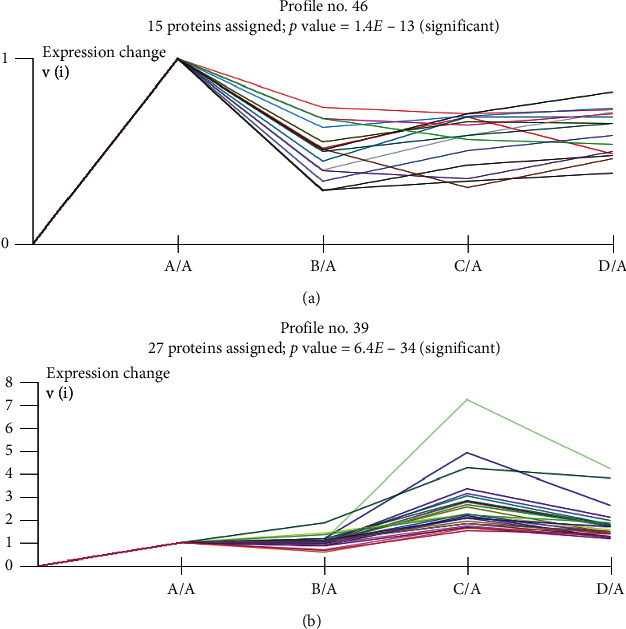
(a) Downregulated protein expression of profile No. 46 and (b) upregulated protein expression of profile No. 39. A: healthy controls; B: NAG group; C: CAG group; D: CAG + ACU group.

According to the above results, a trend analysis of upregulated and downregulated DEPs was performed. A total of 50 model profiles were selected to summarize the expression patterns of proteins to further study the changing DEP trends across the different groups (Figure 6). Among the upregulated DEPs, 4 protein trends were significant (Figure 6(a)), of which the most prominent pattern was profile 39, which contained 27 proteins in total (Figure 7(b)). Among the downregulated DEPs, 4 protein trends were significant (Figure 6(b)), and the most prominent pattern was profile 46, which contained 15 proteins in total (Figure 7(a)).

### 3.7. ELISA Verification

**Figure 8:**
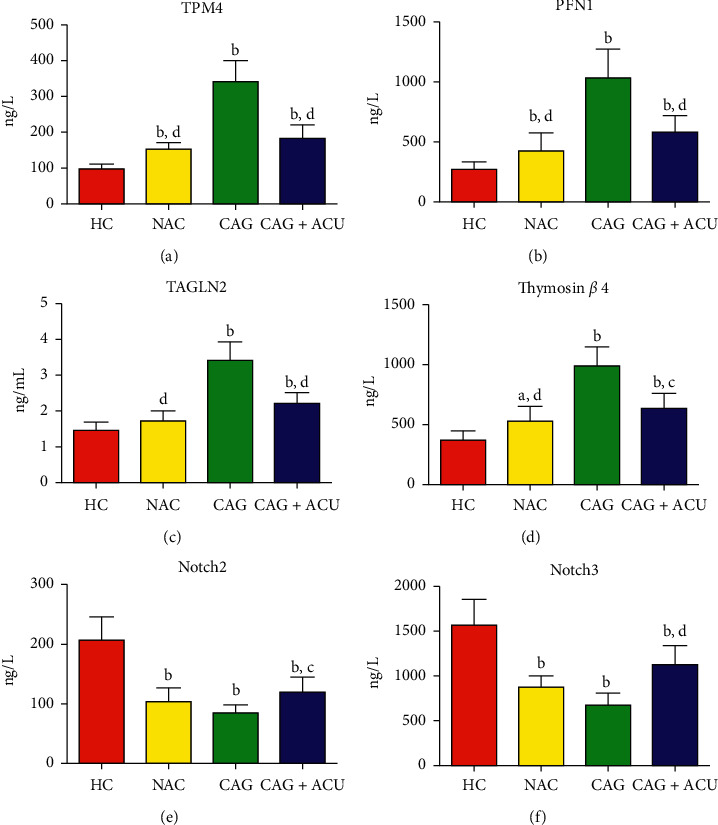
TPM4, PFN1, TAGLN2, thymosin *β* 4, Notch2, and Notch3 levels in 4 groups. Compared to the HC group, ^a^*p* < 0.05 and ^b^*p* < 0.01. Compared to the CAG group, ^c^*p* < 0.05 and ^d^*p* < 0.01.

The results of bioinformatics analysis showed that actin-binding proteins (ABPs) and the Notch signaling pathway-related proteins were closely related to the occurrence and progression of CAG. Six related proteins were selected for ELISA verification in this study, including thymosin beta-4, profilin-1, tropomyosin-4, transgelin-2, Notch2, and Notch3. The trends of the 6 DEPs verified by ELISA were largely similar to those identified via iTRAQ analysis (Table 4). The serum thymosin beta-4, profilin-1, tropomyosin-4, and transgelin-2 protein levels in the CAG group were significantly higher (*p* < 0.01), while the Notch2 and Notch3 protein levels were significantly lower (*p* < 0.05) than in the HC and NAG groups. After acupuncture treatment, the protein contents of thymosin beta-4, profilin-1, tropomyosin-4, and transgelin-2 of the CAG + ACU group were significantly reduced (*p* < 0.05), while the protein contents of Notch2 and Notch3 were significantly increased (*p* < 0.05), compared with the HC and NAG groups (Figure 8).

## 4. Discussion

CAG is the most critical precancerous precursor to gastric cancer. Its pathogenesis is not yet fully elucidated. CAG may be closely related to HP infection, eating habits, bile reflux, and age, genetic, immune, and psychological factors [1]. Our previous studies showed that acupuncture could improve the histopathological changes of gastric mucosa, anxiety, and depression in CAG patients and enhance their living quality [18, 19]. The specific mechanism of CAG's transformation into gastric cancer is not yet understood. Proteomics can be used to study the characteristics of cells, tissues, or biological proteins, such as protein expression levels and post-translational modifications. Understanding the molecular changes in the early stages of the transition from CAG to gastric cancer and is important for the prevention and treatment of gastric cancer.

This study used iTRAQ combined with 2D-LC-MS/MS technology to identify a total of 178 DEPs from the 4 groups. The results of GO enrichment analysis showed that in CAG, the MF mainly involved protein binding, the CC mainly involved cell exosomes, and the BP mainly involved platelet degranulation. In KEGG analysis, the complement system, platelets, chemokines, and other related signaling pathways were activated in NAG, suggesting that NAG involved inflammation and mucosal trauma. In addition to the above signaling pathways, CAG also showed actin cytoskeleton regulation and Notch signaling pathway-related proteins, suggesting that CAG may involve abnormal cytoskeletal changes. PPI analysis also showed that the protein interaction network of CAG involves ABPs, which further confirmed that CAG may involve abnormal cytoskeletal changes and indicated that acupuncture exerted a therapeutic effect on CAG by regulating cytoskeleton-related proteins. Heatmap and trend analyses showed that the DEPs in the NAG group were not significant, and the DEPs in the CAG group were mostly upregulated. After acupuncture treatment, most of the proteins tended to resume normal expression, suggesting that acupuncture simultaneously regulated multiple targets to treat CAG. The trends of the 6 DEPs verified by ELISA were similar to the trends identified by iTRAQ analysis.

This study showed that most of the DEPs upregulated in the sera of CAG patients were related to ABPs, such as thymosin beta-4, profilin-1, tropomyosin-4, and transgelin-2. Actin is the main component of the microfilament system and one of the main components of the cytoskeleton. It plays a vital role in many cell activities, such as muscle contraction, maintenance of cell morphology, movement, division, material transport, and signaling transduction [23]. In addition to actin, there are many other proteins involved in the structure of microfilaments and that regulate microfilament polymerization. These proteins are collectively called ABPs [24, 25]. The malignant transformation of normal epithelial cells is characterized by abnormal changes in the cytoskeleton. ABPs are important targets for regulating cytoskeletal changes [26]. Many studies have found that ABPs are involved in the formation and metastasis of a variety of tumors [27, 28]. However, further studies are needed to verify whether the high levels of ABPs in the serum of CAG patients are related to gastric cancer.

Thymosin beta-4 is one of the main actin regulatory molecules in the human body. It plays an important role in regulating actin microfilament assembly, cell movement and migration, inflammation, damage repair, and other biological activities [29]. Its roles in cornea repair, skin healing, and tumorigenesis have aroused more attention [30–33]. Studies have shown that thymosin beta-4 promotes tumor metastasis and is highly expressed in pancreatic cancer, esophageal cancer, colon cancer, and gastric cancer [34–37]. A study by Sahin et al. [38] also showed that thymosin beta-4 was overexpressed in the gastric mucosa of patients with gastrointestinal stromal tumors (GISTs). Overexpression of thymosin beta-4 in the gastric mucosa was related to tumor progression and poor prognosis in the GIST patients. Therefore, thymosin beta-4 may be a potential diagnostic marker and prognostic indicator of GIST. Profilin-1 is a kind of low-molecular-weight ABPs that play an important role in regulating the polymerization and depolymerization of actin to ensure the steadiness of the microfilament assembly process [39]. Profilin-1 and thymosin beta-4 strengthen or weaken the polymerization of actin, thereby ensuring smooth microfilament assembly [40]. Profilin-1 imbalance has been found to be related to the occurrence and progression of amyotrophic lateral sclerosis, atherosclerosis, hypertension, and tumors [41–45]. Abnormal expression of profilin-1 is related to gastric cancer tumor invasion, lymph node metastasis, and tumor node metastasis (TNM) staging. Silencing profilin-1 inhibits the proliferation and metastasis of gastric cancer cells by inducing cell cycle arrest [45]. Tropomyosin-4 is an important regulatory protein in cell activity and muscle contraction [46]. Tropomyosin combines with F-actin and ABPs to mediate the interaction between F-actin and ABPs. Thus, it participates in cytoskeleton regulation, as well as cell migration and invasion [47]. Studies have shown that abnormal expression of tropomyosin leads to changes in the cytoskeletal structure, along with related incidences of muscle diseases and malignant tumors [48]. Tropomyosin-4 is abnormally expressed in different tumors and plays different roles in promoting or suppressing cancer [49–51]. As a small-molecule ABP, transgelin-2 is widely expressed in smooth muscle cells as well as various tissues and organs in the body and is significantly expressed in organs, such as the stomach, pancreas, and bone marrow [52]. Transgelin-2 regulates the processes involved in cell proliferation, differentiation, migration, and signal transduction by binding to actin [53] and regulates the activation, differentiation, and phagocytosis of lymphocytes and macrophages by regulating the cytoskeleton of actin, thereby regulating the balance of immunity in the body [54]. Transgelin-2 is abnormally and highly expressed in colorectal cancer, ovarian cancer, kidney cancer, gastric cancer, and other malignant tumors [55–57]. In addition, studies have shown that trasgenlin-2 regulates the expression levels of metastasis-related factors and the nuclear factor-kappa B signaling pathway to inhibit cell viability, thereby inhibiting the migration and invasion of cervical cancer cells [58]. At present, no relevant literature has been found on the relationship between acupuncture and ABPs. In this study, compared with the HC group, serum thymosin beta-4, profilin-1, tropomyosin-4, and transgelin-2 were significantly upregulated in CAG patients. However, no significant difference in the levels of those proteins was found between the NAG and the HC groups, suggesting that CAG may involve abnormal changes in the cytoskeleton. The combination of thymosin beta-4, profilin-1, tropomyosin-4, and transgelin-2 expression may be a potential diagnostic marker for CAG. After acupuncture treatment, the expression of above 4 proteins was significantly reduced, suggesting that acupuncture may play a therapeutic role in CAG by reducing ABPs and inhibiting the transformation of CAG into gastric cancer.

This study also showed that Notch signaling pathway-related proteins may be involved in the occurrence and progression of CAG. The Notch family is a highly conserved gene group that involved in cell differentiation, apoptosis, proliferation, and tissue formation [59]. Studies have shown that the Notch signaling pathway plays an important regulatory role in tumor cells and tumor microenvironment, participating in tumor angiogenesis, metastasis, and epithelial-mesenchymal transition in tumor cells, as well as the regulation of drug resistance in tumors [60–63]. Studies have shown that the Notch gene is abnormally activated in many malignant tumors [60–62]. However, some studies indicated that whether the Notch signaling pathway plays an oncogenic or tumor-suppressive role depends on the environment [63, 64]. For example, a study by Hassan et al. [65] showed that Notch3 was overexpressed in non-small-cell lung cancer. However, the expression of Notch3 in small-cell lung cancer was lower than that in the normal lung tissues. The Notch signaling pathway also plays an important role in the tumorigenesis and progression of gastric cancer. Studies have shown that activation of Notch1 and Notch2 in gastric cancer tissues aggravates gastric cancer progression [66, 67]. Inhibition of the Notch signaling pathway enhances the sensitivity of gastric cancer cells to chemotherapy [68]. Expression of Notch1 and Notch2 decreases after gastric cancer resection [69]. In contrast, Bauer et al. [70] showed that patients with high Notch1 expression in early gastric cancer had relatively high survival rates, and thus, Notch1 might play a role in tumor suppression in the early stage of gastric cancer. Previous studies have not examined the Notch signaling pathway-related proteins and CAG. The results of this study indicated that Notch2 and Notch3 protein contents were significantly downregulated in CAG patients. However, no Notch1 abnormalities were found. After acupuncture treatment, the protein contents of Notch2 and Notch3 increased, suggesting that the decrease in serum Notch2 and Notch3 in CAG patients may be related to CAG progression, and acupuncture may treat CAG by increasing Notch2 and Notch3 protein expression.

Collectively, this study utilized iTRAQ technology to conduct exploratory research on serum DEPs in CAG patients vs. healthy controls, as well as in CAG patients after acupuncture treatment. ABPs and Notch signaling pathway-related proteins may play an important role in the occurrence and progression of CAG and may be potential diagnostic markers for CAG. Moreover, results suggest that acupuncture regulated ABPs and proteins related to the Notch signaling pathways in CAG patients. This effect may be an important therapeutic mechanism by which acupuncture treats CAG. Further studies are needed to verify whether these DEPs can be used as diagnostic markers for CAG and whether they are targets of CAG acupuncture treatment.

## 5. Conclusion

Our data presented a valuable resource for diagnosis and treatment for CAG. Acupuncture can modulate multiple abnormal DEPs, such as ABPs and proteins related to the Notch signaling pathways in CAG patients.

## Figures and Tables

**Table 1:** Baseline characteristic.

Clinical parameters	Healthy controls *n* = 8	NAG group *n* = 8	CAG group *n* = 8	*p* value
Age (years)	60.50 ± 4.90	60.13 ± 3.91	62.63 ± 3.66	0.451^a^
Sex (male/female)	3/5	4/4	3/5	0.842^c^
Body mass index	23.35 ± 0.85	23.82 ± 0.88	22.79 ± 0.81	0.697^a^
Smoker (+/−)	2/6	1/7	2/6	0.777^c^
Disease duration (years)	—	5.63 ± 0.88	10.50 ± 1.54	0.014^b^
*Medications (+/*−)				
** **Antibiotics	—	3/5	2/6	0.590^c^
** **Antacids	—	1/7	2/6	0.522^c^
** **PPIs	—	3/5	2/6	0.590^c^

Data are presented as number (mean ± standard deviation). There was no significant difference in sex, age, BMI, and smoking between the three groups. There was no significant difference in medications between NAG and CAG groups. There was a significant difference in disease duration between NAG and CAG groups (*p* < 0.05), ^a^*p* value by variance analysis test. ^b^*p* value by independent samples *t-*test. ^c^*p* by *χ*^2^ test.

**Table 2:** Clinical features of CAG patients before and after acupuncture treatment.

Outcome measures	Before acupuncture treatment *n* = 8	After acupuncture treatment *n* = 8	*p* value
*Clinical symptoms*			
** **TCM syndrome scores	62.13 ± 6.77	28.38 ± 2.30	0.000^a^
** **SF-36 scores	108.69 ± 1.78	116.53 ± 2.07	0.000^a^
** **SDS scores	36.44 ± 1.47	30.44 ± 0.50	0.001^a^
** **SAS scores	35.50 ± 1.07	31.56 ± 0.58	0.008^a^
*Histopathologicalobservation*			
** **Atrophy of gastric mucosa (−/+/++/+++)	0/2/6/0	2/4/2/0	0.037^b^
** **Gastric intestinal metaplasia (−/+/++/+++)	0/4/4/0	4/3/1/0	0.025^b^

Atrophy of gastric mucosa: (−): There was no decrease in the number of inherent glands; (+): The number of inherent glands decreased by no more than 1/3 of the original glands; (++): The number of inherent glands decreased between 1/3 and 2/3 of the original glands; (+++): The number of inherent glands decreased by more than 2/3, with only a few remaining glands or even complete disappearance. Gastric intestinal metaplasia: (−): There was no intestinal metaplasia in gastric mucosa; (+): The area of intestinal metaplasia occupies less than 1/3 of the total area; (++): The area of intestinal metaplasia occupies less than 2/3 of the total area; (+++): The area of intestinal metaplasia occupies more than 2/3 of the total area. TCM: Traditional Chinese Medicine, SF-36: the MOS item short from health survey, SAS: Self-Rating Anxiety Scale, SDS: Self-Rating Depression Scale. ^a^*p* value by paired samples t-test, and ^b^*p* value by Kruskal–Wallis test.

**Table 3:** Differentially expressed proteins in 4 groups.

Group	Upregulation	Downregulation
B vs. A	27	48
C vs. A	75	31
C vs. B	82	28
C vs. D	30	33

The threshold was protein difference fold, which reached 1.4-fold (ratio ≥ 1.4 or ≤ 0.714). A: healthy controls; B: NAG group; C: CAG group; D: CAG + ACU group.

**Table 4:** Selected six serum differential proteins quantitative results in iTRAQ and ELISA analyses.

Gene name	Swissprot number	iTRAQ	ELISA
TPM4	P67936	1 : 0.98 : 3.37 : 2.10	1 : 1.56 : 3.46 : 1.81
TMSB4X	P62328	1 : 1.18 : 4.94 : 2.64	1 : 1.45 : 2.75 : 1.76
TAGLN2	P37802	1 : 0.96 : 3.16 : 1.95	1 : 1.17 : 2.31 : 1.50
PFN1	P07737	1 : 1.03 : 2.24 : 1.36	1 : 1.57 : 3.79 : 2.12
NOTCH2	Q04721	1 : 0.94 : 0.68 : 1.03	1 : 0.50 : 0.42 : 0.58
NOTCH3	M0R3C9	1 : 0.81 : 0.51 : 0.96	1 : 0.56 : 0.44 : 0.72

TPM4: tropomyosin4, TMSB4X: thymosin beta-4, TAGLN2: transgelin-2, PFN1: profilin-1, NOTCH2: Notch2, NOTCH3: Notch3.

## Data Availability

All data are available upon reasonable request to the corresponding authors.

## Abbreviations

DEPs: Differentially expressed proteins
iTRAQ: Isobaric tags for relative and absolute quantitation
CAG: Chronic atrophic gastritis
HC: Healthy volunteers
NAG: Chronic nonatrophic gastritis
CAG + ACU: CAG patients who underwent acupuncture treatment
2D-LC-MS/MS: Two-dimensional liquid chromatography-tandem mass spectrometry
ELISA: Enzyme-linked immunosorbent assay
HP: *Helicobacter pylori*
OLGA: Operative link for gastritis assessment
RN12: Zhongwan
PC6: Neiguan
ST36: Zusanli
FASP: Filter-aided sample preparation
HPLC: Offline high-performance liquid chromatography
GO: Gene Ontology
KEGG: Kyoto Encyclopedia of Genes and Genomes
BMI: body mass index
MF: molecular function
CC: cellular component
BP: biological process
PPI: Protein-protein interaction
TPM4: Tropomyosin-4
TMSB4X: Thymosin beta-4
TAGLN2: Transgelin-2
PFN1: Profilin-1.

## Supplementary Materials

Supplementary Materials Additional file 1: upregulated or downregulated ratio of proteins in different groups. All upregulated (ratio ≥ 1.4) and downregulated proteins (ratio ≤ 0.714) in the groups of HC vs. NAG, HC vs. CAG, and CAG vs. CAG + ACU are presented.
