# Supplementary material for: Acupuncture Regulates Serum Differentially Expressed Proteins in Patients with Chronic Atrophic Gastritis: A Quantitative iTRAQ Proteomics Study
Source: Evid Based Complement Alternat Med. 2021 Jun 14;2021:9962224. doi: 10.1155/2021/9962224 (PMC8219412; doi:10.1155/2021/9962224)
Supplement: Supplementary Materials — Additional file 1: upregulated or downregulated ratio of proteins in different groups. All upregulated (ratio ≥ 1.4) and downregulated proteins (ratio ≤ 0.714) in the groups of HC vs. NAG, HC vs. CAG, and CAG vs. CAG + ACU are presented. [file 9962224.f1.doc]

**Additional file 1: Upregulated or downregulated ratio of proteins in difffferent groups**

| Swissprot number | Protein name information | Molecular  weight  （kDa） | Coverage | HC vs. NAG | HC vs. CAG | CAG vs. CAG+ACU |
| --- | --- | --- | --- | --- | --- | --- |
| A8K2W3 | cDNA FLJ78516 | 47.1 | 1.9 | 1.097 | 7.266 | 4.233 |
| Q6ZW64 | cDNA FLJ41552 fis, highly similar to Protein Tro alpha1 H | 53.3 | 40.7 | 1.463 | 4.997 | 6.706 |
| P62328 | Thymosin beta-4 | 5.0 | 29.6 | 1.179 | 4.939 | 2.642 |
| P01009 | Alpha-1-antitrypsin | 46.7 | 40.4 | 1.866 | 4.275 | 3.812 |
| P35527 | Keratin, type I cytoskeletal 9 | 62.0 | 3.7 | 2.037 | 3.631 | 0.935 |
| P67936 | Tropomyosin alpha-4 chain | 28.5 | 20.6 | 0.984 | 3.365 | 2.104 |
| P37802 | Transgelin-2 | 22.4 | 24.1 | 0.960 | 3.160 | 1.953 |
| A8K7I4 | Calcium-activated chloride channel regulator 1 | 100.2 | 1.8 | 1.095 | 3.111 | 5.059 |
| Q5T123 | SH3 domain-binding glutamic acid-rich-like protein SH3 | 9.4 | 42.1 | 1.109 | 3.056 | 1.804 |
| H6VRF8 | Keratin 1 | 66.0 | 8.2 | 1.801 | 3.001 | 1.178 |
| B4E190 | cDNA FLJ57770, moderately similar to ADP-ribosylation factor 3 | 16.1 | 10.4 | 1.152 | 2.837 | 1.783 |
| Q86TQ3 | Similar to filamin C | 18.9 | 22.0 | 0.950 | 2.785 | 1.722 |
| K7EL66 | Microtubule-associated protein RP/EB family member 2 | 15.0 | 6.0 | 0.995 | 2.780 | 1.680 |
| Q5HYB6 | Putative uncharacterized protein DKFZp686J1372 | 27.2 | 16.4 | 0.966 | 2.663 | 1.758 |
| Q14019 | Coactosin-like protein | 15.9 | 22.5 | 1.026 | 2.555 | 1.400 |
| P13645 | Keratin, type I cytoskeletal 10 | 58.8 | 7.5 | 2.580 | 2.514 | 1.937 |
| P02671 | Fibrinogen alpha chain | 94.9 | 25.8 | 1.425 | 2.441 | 2.565 |
| A8KA84 | cDNA FLJ78682, highly similar to Homo sapiens 2'-5'-oligoadenylate synthetase 3 | 121.1 | 0.8 | 1.333 | 2.305 | 2.819 |
| Q86YW5 | Trem-like transcript 1 protein | 32.7 | 5.1 | 1.411 | 2.255 | 1.541 |
| P07737 | Profilin-1 | 15.0 | 44.3 | 1.032 | 2.238 | 1.357 |
| D6REL8 | Fibrinogen beta chain | 31.2 | 20.2 | 1.334 | 2.221 | 1.871 |
| P02647 | Apolipoprotein A-I | 30.8 | 75.7 | 1.835 | 2.186 | 3.052 |
| B7Z7M2 | cDNA FLJ51564, highly similar to Pregnancy zone protein | 15.0 | 46.3 | 0.838 | 2.171 | 1.196 |
| G3V226 | Calmodulin | 8.9 | 15.8 | 0.958 | 2.093 | 1.462 |
| Q71V99 | Peptidyl-prolyl cis-trans isomerase | 18.0 | 24.4 | 1.083 | 2.065 | 1.682 |
| Q5TCU6 | Talin-1 | 257.9 | 1.9 | 0.970 | 2.056 | 1.184 |
| P02679 | Fibrinogen gamma chain | 51.5 | 10.8 | 1.259 | 2.030 | 2.049 |
| P07996 | Thrombospondin-1 | 129.3 | 36.0 | 1.397 | 1.998 | 1.399 |
| P02776 | Platelet factor 4 | 10.8 | 42.6 | 1.630 | 1.948 | 1.587 |
| K7EIW7 | Platelet glycoprotein VI | 13.0 | 8.6 | 1.089 | 1.942 | 1.372 |
| H0YIN9 | Keratin, type II cytoskeletal 5 | 22.0 | 4.6 | 1.423 | 1.903 | 1.137 |
| G3V1A4 | Cofilin 1 | 16.8 | 9.4 | 1.070 | 1.821 | 1.467 |
| C9JL85 | Myotrophin | 5.7 | 32.7 | 1.106 | 1.820 | 1.259 |
| B4E3I0 | cDNA FLJ55017, highly similar to Caldesmon | 35.4 | 6.1 | 1.047 | 1.811 | 1.431 |
| H0YAP2 | Polyadenylate-binding protein 1 | 15.1 | 7.6 | 0.918 | 1.708 | 1.249 |
| Q0KKI6 | Immunoblobulin light chain | 24.0 | 44.8 | 0.909 | 1.674 | 2.087 |
| B4DW08 | cDNA FLJ50886, highly similar to Aconitate hydratase | 54.4 | 1.2 | 0.678 | 1.673 | 1.246 |
| Q86XU5 | MYH9 protein | 158.7 | 0.5 | 1.267 | 1.664 | 1.143 |
| H0YNC7 | Tropomyosin alpha-1 chain | 25.6 | 14.8 | 0.861 | 1.658 | 1.158 |
| P10124 | Serglycin | 17.6 | 12.0 | 1.219 | 1.652 | 1.343 |
| B7Z8A9 | cDNA FLJ51166, highly similar to Matrix metalloproteinase-9 | 40.8 | 4.6 | 1.406 | 1.636 | 1.049 |
| Q04756 | Hepatocyte growth factor activator | 70.6 | 33.9 | 0.585 | 1.625 | 1.483 |
| P01605 | Ig kappa chain V-I region Lay | 11.8 | 8.3 | 0.950 | 1.623 | 2.072 |
| H0YID2 | Adenylate kinase isoenzyme ^1^ | 5.8 | 19.6 | 0.794 | 1.603 | 2.367 |
| B4E3J7 | cDNA FLJ51203, highly similar to Thrombospondin-1 | 120.1 | 32.2 | 1.227 | 1.601 | 1.275 |
| B7ZKS9 | NLRP3 protein | 115.9 | 0.9 | 1.296 | 1.580 | 1.835 |
| P01833 | Polymeric immunoglobulin receptor | 83.2 | 3.1 | 1.031 | 1.574 | 1.346 |
| M0R2W8 | N-acetylmuramoyl-L-alanine amidase | 15.5 | 18.6 | 1.075 | 1.570 | 1.008 |
| P40197 | Platelet glycoprotein V | 60.9 | 27.1 | 1.299 | 1.570 | 1.320 |
| P05155 | Plasma protease C1 inhibitor | 55.1 | 32.2 | 0.419 | 1.555 | 1.425 |
| Q9P273 | Teneurin-3 | 300.8 | 0.3 | 1.209 | 1.544 | 1.051 |
| E9PNQ9 | Tumor protein D53 | 10.5 | 7.6 | 1.287 | 1.527 | 1.305 |
| E5RGE1 | 14-3-3 protein zeta/delta | 5.9 | 42.3 | 0.925 | 1.514 | 1.079 |
| Q68CK4 | Leucine-rich alpha-2-glycoprotein | 38.1 | 46.1 | 0.628 | 1.509 | 1.387 |
| F6KPG5 | Albumin | 66.5 | 41.4 | 1.444 | 1.504 | 1.379 |
| A2KBB9 | Anti-(ED-B) scFV | 25.1 | 19.3 | 0.941 | 1.503 | 1.919 |
| Q15849 | Urea transporter 2 | 101.1 | 0.7 | 0.989 | 1.503 | 1.018 |
| B7Z4X6 | cDNA FLJ51012, highly similar to Plasminogen activator inhibitor 1 | 38.0 | 2.7 | 1.215 | 1.492 | 1.147 |
| P10720 | Platelet factor 4 variant | 11.5 | 45.2 | 1.400 | 1.489 | 1.207 |
| A6NML8 | Diaphanous homolog 2 | 124.8 | 1.0 | 1.844 | 1.478 | 1.813 |
| Q8WTQ7 | G protein-coupled receptor kinase 7 | 62.2 | 2.9 | 3.243 | 1.475 | 3.011 |
| Q6P2N0 | Myosin light chain kinase | 16.8 | 7.2 | 1.115 | 1.470 | 1.215 |
| M0R1V7 | Ubiquitin-60S ribosomal protein L40 | 7.1 | 14.3 | 1.025 | 1.468 | 1.611 |
| P10599 | Thioredoxin | 11.7 | 21.0 | 0.948 | 1.455 | 1.418 |
| S6B2A1 | IgG L chain | 20.4 | 22.3 | 0.922 | 1.452 | 1.846 |
| P61626 | Lysozyme C | 16.5 | 42.6 | 1.121 | 1.449 | 1.338 |
| A2NB46 | Cold agglutinin FS-2 L-chain | 11.9 | 31.2 | 1.030 | 1.437 | 1.630 |
| B2RBZ5 | cDNA, FLJ95778, highly similar to Homo sapiens serpin peptidase inhibitor, clade A | 50.6 | 35.6 | 1.399 | 1.437 | 1.376 |
| A7E2F7 | CAP-GLY domain containing linker protein 2 | 111.7 | 0.9 | 1.071 | 1.430 | 1.520 |
| H7C229 | Cytoplasmic FMR1-interacting protein 2 | 110.4 | 1.0 | 1.334 | 1.417 | 1.599 |
| P05090 | Apolipoprotein D | 21.3 | 29.6 | 1.375 | 1.414 | 1.462 |
| Q6MZQ6 | Putative uncharacterized protein DKFZp686G11190 | 52.0 | 20.6 | 0.826 | 1.408 | 1.667 |
| A2J1M4 | Rheumatoid factor RF-ET7 | 10.9 | 23.5 | 0.903 | 1.407 | 1.417 |
| L8EAW6 | Alternative protein DYZ1L9 | 7.0 | 9.8 | 1.128 | 1.401 | 1.324 |
| P01781 | Ig heavy chain V-III region GAL | 12.7 | 17.2 | 0.802 | 1.400 | 1.794 |
| Q6P089 | IGH@ protein | 52.0 | 36.0 | 0.400 | 0.704 | 0.839 |
| B7ZLM6 | X-prolyl aminopeptidase 2 | 75.6 | 1.5 | 0.736 | 0.701 | 0.724 |
| J3KQY4 | Transcription elongation factor A protein-like 4 | 5.8 | 22.0 | 1.123 | 0.701 | 0.695 |
| S6BGD6 | IgG L chain | 24.8 | 45.5 | 0.506 | 0.701 | 0.820 |
| Q7Z443 | Polycystic kidney disease protein 1-like 3 | 195.8 | 0.8 | 0.783 | 0.699 | 0.721 |
| B7Z539 | cDNA FLJ56954, highly similar to Inter-alpha-trypsin inhibitor heavy chain H1 | 72.1 | 42.5 | 0.785 | 0.691 | 0.769 |
| P01859 | Ig gamma-2 chain C region | 35.9 | 25.8 | 0.629 | 0.689 | 0.731 |
| P01880 | Ig delta chain C region | 42.2 | 32.3 | 0.515 | 0.686 | 0.483 |
| Q9UL82 | Myosin-reactive immunoglobulin light chain variable region | 11.4 | 10.3 | 0.444 | 0.683 | 0.684 |
| M0R3C9 | Neurogenic locus notch homolog protein 3 | 134.2 | 1.8 | 0.935 | 0.683 | 1.029 |
| E9PH42 | Receptor-type tyrosine-protein phosphatase U | 161.8 | 0.8 | 0.964 | 0.678 | 0.727 |
| Q8N589 | PMAIP1 protein | 14.9 | 8.8 | 0.550 | 0.660 | 0.648 |
| H7C3N9 | Leucine-rich repeat flightless-interacting protein 2 | 21.0 | 4.4 | 1.115 | 0.651 | 0.660 |
| O60747 | Putative G-binding protein | 65.4 | 1.8 | 1.180 | 0.644 | 0.827 |
| L8E853 | von Willebrand factor | 298.2 | 29.8 | 0.674 | 0.641 | 0.702 |
| K7EQ47 | Spermidine synthase | 14.4 | 6.0 | 1.263 | 0.638 | 0.843 |
| P04196 | Histidine-rich glycoprotein | 59.5 | 39.4 | 0.827 | 0.592 | 0.794 |
| H0Y5V5 | Serine/threonine-protein kinase N2 | 24.3 | 3.8 | 1.090 | 0.591 | 0.791 |
| Q71MF9 | Putative uncharacterized protein FP6679 | 10.8 | 23.4 | 1.553 | 0.588 | 0.869 |
| Q6GMW4 | IGL@ protein | 24.8 | 44.2 | 0.396 | 0.585 | 0.712 |
| Q5NV90 | V2-17 protein | 10.4 | 21.7 | 0.500 | 0.584 | 0.652 |
| B7Z795 | cDNA FLJ57637, highly similar to Liver carboxylesterase 1 | 47.7 | 4.2 | 0.677 | 0.564 | 0.536 |
| H7C226 | Lutropin-choriogonadotropic hormone receptor | 19.8 | 5.1 | 2.101 | 0.522 | 0.680 |
| Q1HP67 | Lipoprotein, Lp(A) | 226.4 | 36.8 | 0.729 | 0.521 | 1.086 |
| Q04721 | Neurogenic locus notch homolog protein 2 | 265.2 | 0.5 | 0.814 | 0.505 | 0.955 |
| J3KPV0 | Peroxisomal targeting signal 1 receptor | 45.9 | 3.4 | 0.335 | 0.503 | 0.585 |
| P01860 | Ig gamma-3 chain C region | 41.3 | 38.2 | 0.284 | 0.422 | 0.477 |
| P00748 | Coagulation factor XII | 67.7 | 44.1 | 2.677 | 0.374 | 1.010 |
| J3QS23 | Cerebellin-2 | 12.2 | 7.4 | 0.392 | 0.351 | 0.500 |
| H7C3R1 | Nuclear receptor subfamily 2 group C member 2 | 19.8 | 5.8 | 0.289 | 0.336 | 0.382 |
| G1AUC5 | Protein phosphatase inhibitor 2-like protein | 23.1 | 7.3 | 0.512 | 0.302 | 0.459 |
